# Characterization of Modified PVDF Membranes Using Fourier Transform Infrared and Raman Microscopy and Infrared Nanoimaging: Challenges and Advantages of Individual Methods

**DOI:** 10.1021/acsomega.4c01197

**Published:** 2024-05-31

**Authors:** Matěj Kmetík, Ivan Kopal, Martin Král, Marcela Dendisová

**Affiliations:** Department of Physical Chemistry, University of Chemistry and Technology Prague, Technická 5, Prague 6 166 28, Czech Republic

## Abstract

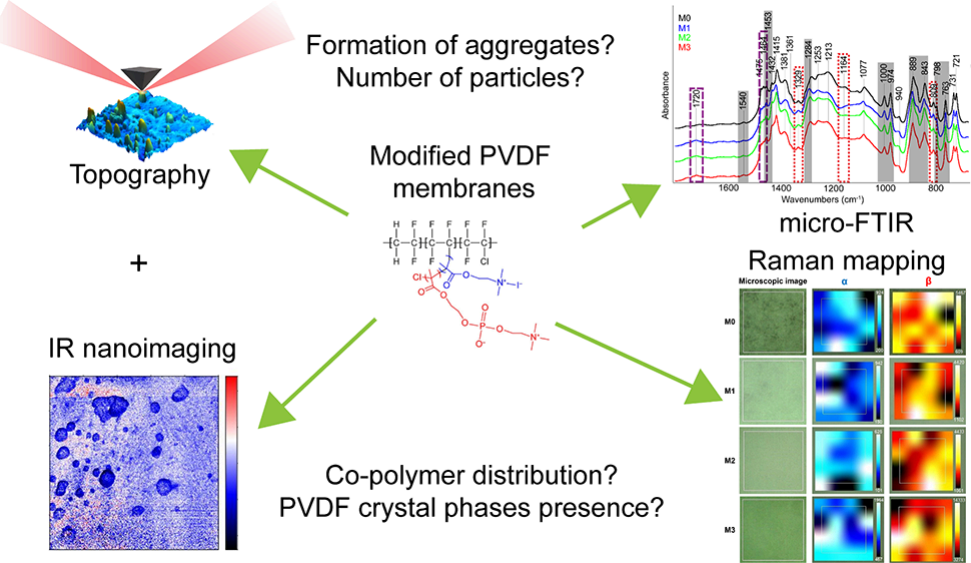

Polymer materials are integral to diverse scientific fields, including chemical engineering and biochemical research, as well as analytical and physical chemistry. This study focuses on the characterization of modified poly(vinylidene fluoride) (PVDF) membranes from both physical and chemical perspectives. Unfortunately, current surface characterization methods face various challenges when simultaneously measuring diverse material properties such as morphology and chemical composition. Addressing this issue, we introduce infrared scattering scanning near-field optical microscopy (IR-sSNOM), a modern technique with the ability to overcome limitations and provide simultaneous topographical, mechanical, and chemical information. We demonstrate the capabilities of IR-sSNOM for investigation of four samples of PVDF membranes modified with 2-(methacryloyloxyethyl)trimethylammonium iodide and/or methacryloyloxyethyl phosphorylcholine in various ratios. These membranes, desirable for their specific properties, represent a challenging material for analysis due to their thermal instability and mechanical vulnerability. Employing Fourier transform infrared (FTIR) microscopy, IR-sSNOM, and Raman microscopy, we successfully overcame these challenges by carefully selecting the experimental parameters and performing detailed characterization of the polymer samples. Valuable insights into morphological and chemical homogeneity, the abundance of modifying side chains, and the distribution of different crystal phases of PVDF were obtained. Most notably, the presence of modifying side chains was confirmed by FTIR microscopy, the Raman spectral mapping revealed the distribution of crystalline phases of the studied polymer, and the IR-sSNOM showed the abundance of chemically diverse aggregates on the surface of the membranes, thanks to the unique nanometer-scale resolution and chemical sensitivity of this technique. This comprehensive approach represents a powerful toolset for characterization of polymeric materials at the nano- and microscale. We believe that this methodology can be applied to similar samples, provided that their thermal stability is considered, opening avenues for detailed exploration of physical and chemical properties in various scientific applications.

## 1 Introduction

In today’s chemical practice, polymer materials form an undoubtedly important group of substances that are crucial in various chemical and material fields, such as chemical engineering,^1^ biochemical research,^2^ and also analytical and physical chemistry.^3^ Despite increasing numbers of reports dealing with the health-endangering properties of polymers,^4^ especially microplastics,^5^ it is hardly imaginable to fully exclude polymers from the contemporary scientists’ field of view. Particular attention is paid to polymer membranes as they play significant roles in chemical technology processes,^6^ food industry, medicinal-related applications,^7^ and environmental chemistry.^8^

Hand in hand with the increasing importance of membranes comes the necessity of their proper characterization from both physical and chemical points of view. Although the currently well-established methods of surface characterization, such as electron microscopy, atomic force microscopy (AFM), X-ray spectroscopy, vibrational spectroscopy, and others,^9−14^ are often sufficient, they all encounter a significant issue when the requirement of simultaneous measurement of several properties (e.g., morphology and chemical composition) arises. This issue can be overcome by the implementation of a modern technique, infrared scattering scanning near-field optical microscopy (IR-sSNOM). This is representative of scanning near-field optical microscopies (SNOM), whose principle was first introduced by Betzig and Trautman in 1992^15^ and put in relation with vibrational absorption by Knoll and Keilmann.^16^ Nevertheless, the first idea of how to overcome Abbe's diffraction limit^17^ was presented already in 1928 by Synge.^18^ However, technical possibilities enabled the actual development of Synge's idea several decades later. In a scattering-type SNOM (sSNOM) setup, a sharp metallic AFM tip is illuminated with a laser beam, and the enhanced near-field signal from the close vicinity of the apex of the tip is collected.^19,20^ One of the main advantages of IR-sSNOM is the simultaneous acquisition of topography (an AFM image) and mechanical and chemical properties through optical channels carrying information about the reflective (amplitude) and absorptive (phase) properties of the sample. For further details about the principles of this technique, kindly refer to the cited literature.^21−26^ The schematic experimental setup can be seen in Figure S1 in the Supporting Information. Nowadays, IR-sSNOM plays a priceless role in numerous scientific fields including, for example, semiconductor research,^27−29^ physical chemistry,^30^ biophysical chemistry,^31^ or the previously mentioned polymer-related application fields.^32,33^

In this study, we aim to demonstrate the capabilities of the IR-sSNOM technique when investigating complex membrane samples represented by a series of poly(vinylidene fluoride) (PVDF) samples modified with 2-(methacryloyloxyethyl)trimethylammonium iodide (QDMA) and/or methacryloyloxyethyl phosphorylcholine (MPC). These membranes, much more desirable in current practice due to their specific properties, such as high biodegradability, represent a typical example of “hard-to-handle” samples, mainly due to their thermal instability and mechanical vulnerability. These membranes show promising abilities for water treatment; therefore, characterization of their properties may have impact onto the utilization of these membranes into the environmental fields.^34^ Using the combined advantages of FTIR microscopy, IR-sSNOM, and Raman microscopy, we were able to overcome most of these issues and performed a detailed characterization of these polymer samples. As a result, we managed to obtain several valuable characteristics of the membranes, such as information about morphological and chemical homogeneity, abundance of modifying side chains, and distribution of different crystal phases of PVDF.

## 2 Materials and Methods

### 2.1 Materials

**Figure 1:**
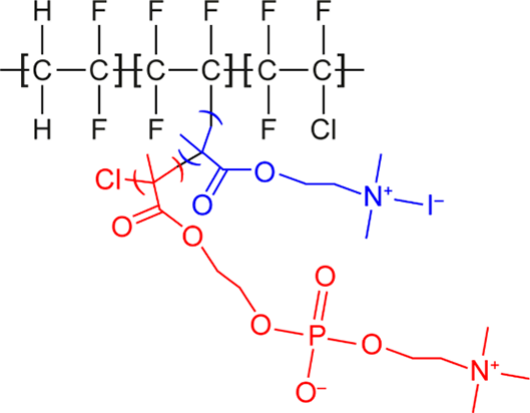
Chemical structure of modified PVDF. The QDMA additive is displayed in blue, and the MPC is in red.

The novel polymer samples were prepared by the group of Han and introduced in the article.^34^ In our study, we are using exactly the same samples as in the mentioned reference. For detailed information about the chemicals used and the synthesis of the membranes, we would like to refer to the article by Han et al.^34^ Briefly, the first step in synthesizing the polymer is to mix pure and modified PVDF in a dimethyl sulfoxide solution. Subsequently, the mixture is spread on a glass plate with nonwoven material from polyethylene terephthalate (PET), which is then immersed in deionized water to remove the solvent. This immersion method in deionized water is called non-solvent-induced phase separation (NIPS).^35,36^ The layer undergoes a process of poresation, and the membranes are subsequently dried at elevated temperatures.^35,36^ QDMA and MPC are commonly added to the mentioned mixture because of their ability to improve the membrane properties (microbial resistivity, biocompatibility, and resistivity to oil, bacteria, and protein pollution). It has been shown that these modifications can prolong the life of the membranes.^37−40^ These modifying substances were also incorporated into the membranes characterized in this work. The structure of the PVDF polymer chain with the modifying side chains is illustrated in Figure 1.

In total, four samples were studied, which differed in the presence and ratio of added MPC and QDMA groups collectively denoted as PVDF-A, PVDF-B, and PVDF-C (Table 1). The M0 membrane contained no additions, while the M3 membrane included the highest amount of MPC.

**Table 1:** Composition of the Studied PVDF Samples^a^

Sample	PVDF (g)	Added polymer	MPC (mol %)	QDMA (mol %)
M0	3			
M1	2.82	PVDF-A (0.18 g)	0	100
M2	2.82	PVDF-B (0.18 g)	16	84
M3	2.82	PVDF-C (0.18 g)	72	28

a The added polymers are labeled according to the molar fraction of the additive (PVDF-A, PVDF-B, and PVDF-C).

### 2.2 FTIR Microspectroscopy

The Micro-FTIR spectrometer used for the measurements was an iN10 infrared microscope (Thermo Fisher Scientific, USA). The microscope operated as a module and was added to the FTIR spectrometer iS10 from the same company. This instrument uses a heated ceramic rod as a source of irradiation in the mid-infrared region and is equipped with a nitrogen-cooled MCT (mercury–cadmium–telluride) detector. The whole instrument was controlled by Omnic Picta software (version 1.7.224, Thermo Fisher Scientific, USA), where the primary data processing was also performed. Measurements were performed in reflection mode with 128 scans per measurement and an aperture of 300 × 300 μm. The measurements were carried out in the form of 10 × 10 point maps with a step size of 300 μm. Afterward, all 100 spectra from each sample were averaged with the Omnic Picta software.

### 2.3 Infrared Near-Field Nanoscopic Imaging

The IR-sSNOM measurements were realized using a commercially available neaSNOM microscope (Neaspec, Attocube systems AG, Germany). The microscope was operating in AFM tapping mode. A set of three tunable quantum cascade lasers (QCL, MIRCAT, Daylight Solutions, USA) was used as a monochromatic infrared radiation source, and the scattered light was passed through a Michelson interferometer and detected by a liquid nitrogen-cooled MCT detector. The samples were positioned on a magnetic *x*, *y*, and *z* position table with a range of 100 μm in the *x* and *y* axis directions (minimum step of 0.4 nm) and 2.5 μm in the *z* axis (minimum step of 0.2 nm). For all measurements, Arrow-NCPt AFM silicon tips providing high lateral resolution were used. The Arrow-NCPt tips were manufactured by NanoWorld (nanoworld.com, Switzerland) and were made out of silicon coated with a layer of platinum–iridium alloy. The oscillation frequency of the tip ranges between 240 and 380 kHz (for our measurements, this value was set at 285 kHz), while its motion is recorded using a 790 nm laser. The elasticity constant of this tip is 42 N/m, and the tip radius of curvature is reportedly less than 25 nm. The neaSNOM instrument was equipped with a built-in digital camera (5 MPix resolution) with an optical microscope for observing the position of the tip on the sample. The lateral resolution of the optical system was approximately 800 nm.

The integration time was 13.1 ms per pixel, and the resolution of the displayed images was set to 256 × 256 pixels. All sample surfaces were scanned in an area of 5 × 5 μm. The infrared irradiation source was tuned to the frequency of the known absorption bands (974, 1000, and 1720 cm^–1^) that were selected based on our measurements using Micro-FTIR spectrometry (see Section 3.1). For data processing, we utilized the freely available software Gwyddion (version 2.60.20211114, Czech Republic).^41^ All presented IR-sSNOM images were adjusted by subtracting the plane level; horizontal scratches were corrected, line scratches were smoothed if necessary, and the gradually changing background caused by thermal drift was corrected using the line median of the difference of the matching function. Subsequently, the background was subtracted by a fifth-degree polynomial. The software was subsequently used to select nanoparticles and obtain statistic parameters such as roughness, average size of nanoparticles, and number of nanoparticles per unit of area.

### 2.4 Confocal Raman Microscopy

The modified PVDF membranes were also analyzed using the Raman Microscope InVia (Renishaw, UK). The spectrometer was equipped with a 785 nm diode laser, a thermoelectrically cooled CCD detector, and a grating with a groove density of 1800 lines per mm. The Raman spectra were measured with the laser power of 3 mW (on the sample), while for mapping, the laser power was set at 0.6 mW. The objectives with magnifications of 50 and 100× were used for the “single” spectra collection and mapping, respectively. The spectral resolution was better than 2 cm^–1^, and five accumulations with 30 s of integration time were set up for each “single” spectrum. For spectral mapping, different conditions were used: the number of accumulations was 10 and the integration time was 240 s. The maps were recorded in the area of 4 × 4 points with a step of 10 μm, and the diameter of the laser spot was ca. 5 μm. The instrument is controlled by WiRe (version 4.0, Renishaw, UK), which was also used for baseline correction and processing of the acquired Raman maps.

## 3 Results and Discussion

### 3.1 Micro-FTIR Spectra of the Membranes

First, the membranes were characterized using Micro-FTIR, which was used for the identification of specific bands of PVDF and PVDF modified with added side chains (QDMA and MPC), while some of these bands were later used for a detailed study using the IR-sSNOM nanoimaging (Section 3.2). In the spectra, mostly bands of the main chain of the PVDF were observed; nevertheless, the signal of the side chains was also noticeable. It was confirmed that the membranes contain several copolymers, which exhibit bands at specific wavenumbers and affect the spectral profile of neat PVDF. This observation itself confirms the success of the modification. Also, from the fine differences in the spectra, the presence of α, β, and γ crystalline phases of the PVDF (whose characteristic bands will be described later) could be identified based on the data formerly published in the literature.^42,43^

**Figure 2:**
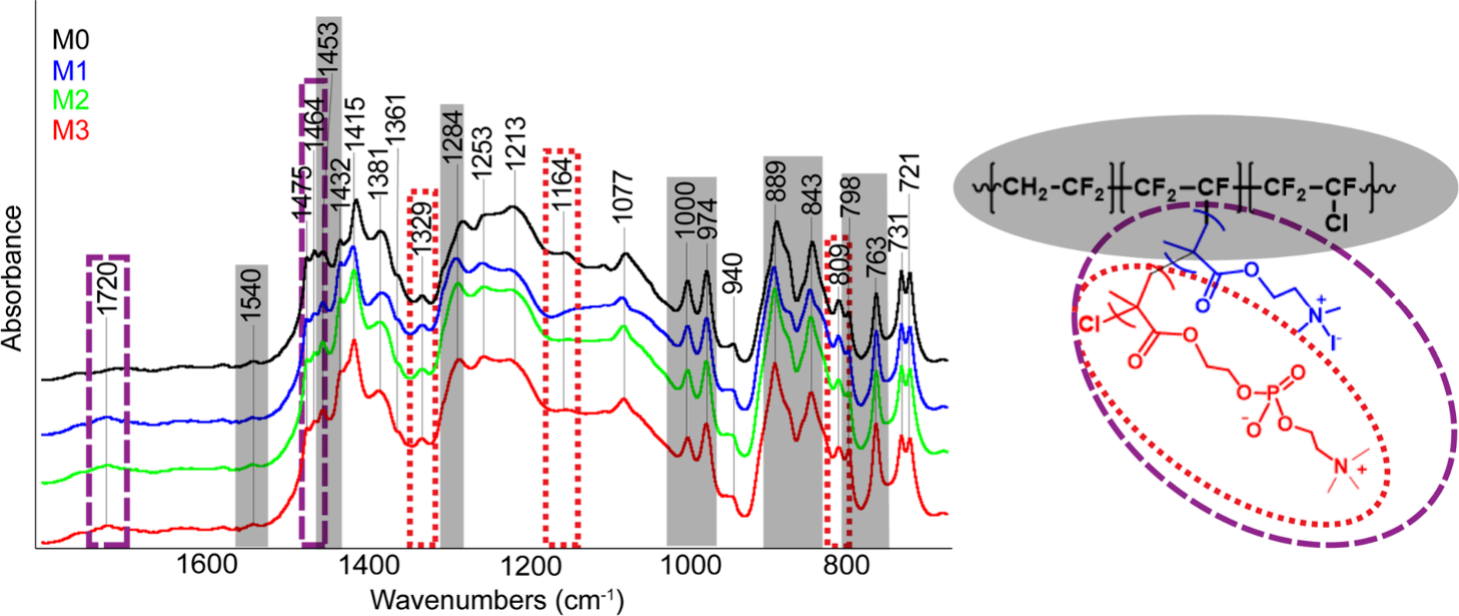
Averaged reflection Micro-FTIR spectra of the analyzed PVDF membrane samples (M0, M1, M2, and M3 from top to bottom). Structure of the polymer with the color assignment (right). The highlighted spectral regions correspond to vibrations of the main PVDF chain (gray), the MPC side chain (red dots), and shared bands for both side chains (violet lines). The spectra are displayed on an offset scale.

From the obtained Micro-FTIR spectra of the samples (Figure 2) the characteristic bands were assigned to functional groups of PVDF, MPC, or QDMA. The assignment of each individual band to a specific part of the polymer chain is demonstrated in Figure 2. The band at 1720 cm^–1^, which belongs to the stretching C=O vibration, originates from the side chains bound to PVDF. The next band at 1540 cm^–1^ was assigned to the skeletal vibration of the CH_2_ group. Although this position is noticeably higher than that normally, we believe that this is caused by the incorporation of these groups into the polymer chain. The 1453 cm^–1^ band was assigned to the stretching vibration of the CH_2_ group next to the electronegative element, so it consists of several overlapping bands. The rest of the bands from this group can probably be assigned to the CH_2_ group from the attached chain, where this group is located between MPC and PVDF. The band at 1284 cm^–1^ originates from the stretching vibration of the CF_2_ group of the PVDF β crystalline phase. The band at 1000 cm^–1^ is assigned to the F–C–Cl skeletal vibration, which is located in the main chain of the PVDF. The band at 974 cm^–1^, which belongs to the α crystalline phase, can be identified as the twisting vibration of the CH_2_ group (this band was selected as one of the representatives of the main chains to investigate the surface homogeneity or coating perfection of the substrate layer by IR-sSNOM). The mode at 889 cm^–1^ is assigned to an antisymmetric stretching C–C vibration. The band at 843 cm^–1^ is characteristic for the β and γ crystalline phases for the CH_2_ group rocking vibration and stretching antisymmetric vibration of the CF_2_ group.^42,43^ The band at 809 cm^–1^, visible as a shoulder, belongs to the γ crystalline phase. A typical vibration for the α phase is 798 cm^–1^ and originates from the vibration of the CH_2_ group. The α phase is also manifested through the rocking vibration located at 763 cm^–1^, which arises from the CF_2_ group. Even though the bands of PVDF in different phases were observed together with bands of additives, due to poor spatial resolution, we are not able to argue about their distribution on the surface of the sample, and this deficiency was surmounted by using IR-sSNOM whose results are described further.

It is apparent that most of the bands are wide, which inevitably leads to the hypothesis that they may be composed of modes originating from several different functional groups. This theory could possibly be confirmed thanks to the Raman spectra, which can be seen and is discussed in Section 3.3. The first complex peak is located at 1464 cm^–1^. This vibrational energy is typical for the CH_2_ group, but in our case, it almost certainly refers to the several types of this group located at different places of the examined polymer structure. This information is important because it is possible to confirm the presence of side chains in the molecule. Although the main contribution of the affected areas originates from the main PVDF chain, the other modes consisting of multiple vibrations assigned to MPC can be distinguishable, e.g., bands at ca. 1329, 1164, and 809 cm^–1^ which are typical for the P=O group, namely, its stretching vibration. The assignment of all vibrations in the Micro-FTIR spectra is summarized in Table S1 in the Supporting Information. The similarity with the Raman spectra can be seen in the Supporting Information in Figure S3.

### 3.2 IR-sSNOM Imaging of PVDF Membranes

**Figure 3:**
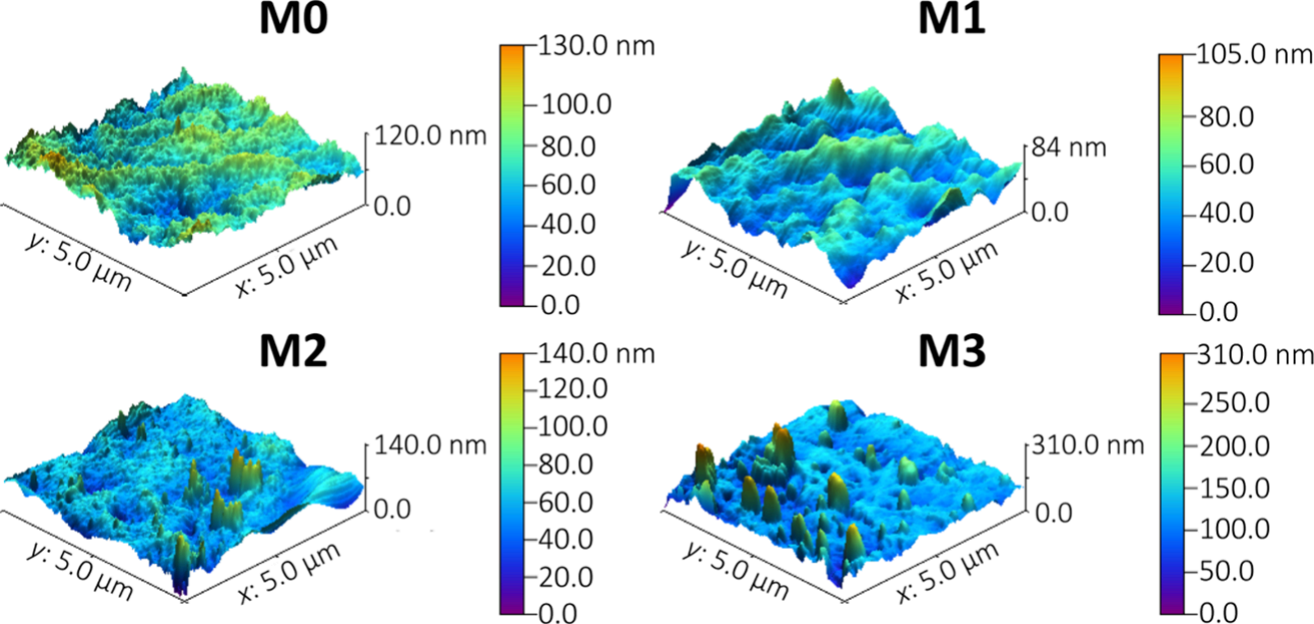
3D AFM images of the surface topography of the studied membranes.

The AFM topography records obtained by the IR-sSNOM technique (Figure 3) revealed the possible formation of aggregates, which can be induced by interlacing the side chains. Based on the topography images, we were able to evaluate several physical properties of the systems, such as the average height of surface features, their number, and average roughness.

**Figure 4:**
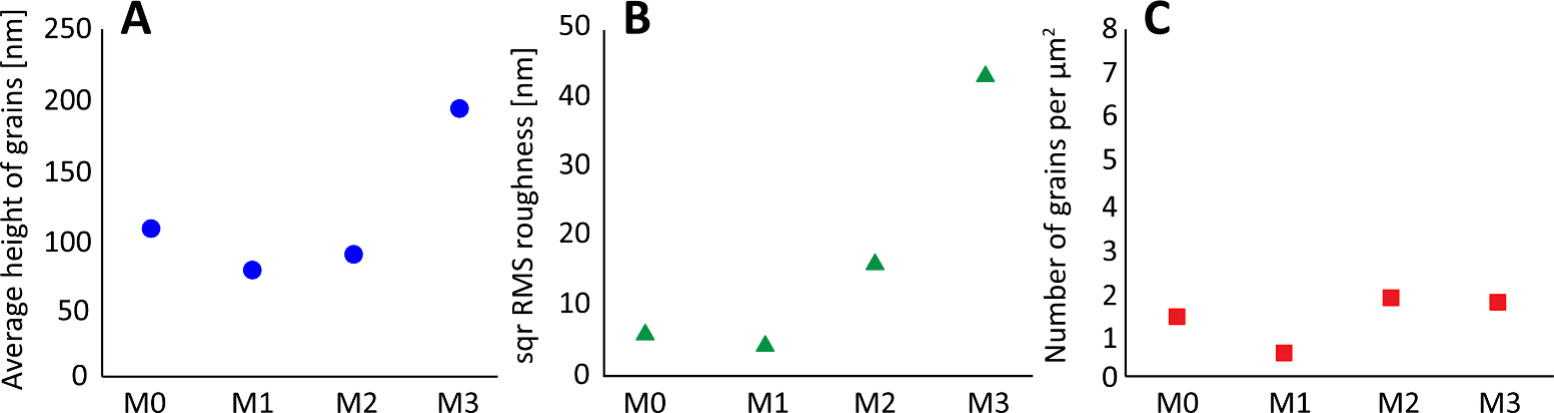
Values of studied statistical surface parameters on PVDF membrane samples: (average grain height (A), square RMS roughness of membrane layers (root mean square roughness) (B), and number of grains per μm^2^ (C)).

The AFM images of the samples’ surface morphology show that some of the membranes are very structured, and the present features are quite abundant in several cases (samples M2 and M3). While the size distribution of the aggregates is sufficiently homogeneous for one particular membrane, it significantly differs between the studied samples (Figure 4). Samples M0 and M1 show no signs of aggregation as their surfaces do not exhibit any significant morphological changes. The highest detected features were on the membrane M3, which reached a height of approximately 300 nm; however, the surroundings of the discussed features are mostly flat, which is in strong contrast with the observations made on the M2 sample. The M3 membrane also has the highest average feature height and the highest roughness. The average height of the features on the M3 membrane surface is about 195 nm. The other membranes exhibited an average high of features of approximately 100 nm. The surface height of the samples ranges from 105 to 310 nm relative to the lowest spot on the image. Overall, it could be stated that the addition of MPC leads to the increased roughness of the surface.

The IR-sSNOM technique is ideal for the detection and identification of chemical entities present on the sample’s surface. In our case, the use of IR-sSNOM enabled the assessment of the overall chemical homogeneity of the membranes’ surface. The employed infrared radiation source was tuned to the frequency of the known vibrational bands that were selected based on the Micro-FTIR measurements. The first two wavenumbers (974 and 1000 cm^–1^ assigned to the CH_2_ vibration of PVDF in the α phase and F–C–Cl skeletal vibration, respectively) were selected because it is highly desired to describe the uniformity and homogeneity of the polymer layer covering the substrate. The last selected irradiation wavenumber (1720 cm^–1^ assigned to the stretching C=O vibration of both additives) was selected because the features exhibiting a higher response to this wavenumber can contain increased amounts of QDMA and MPC, which may affect their other physicochemical properties.

**Figure 5:**
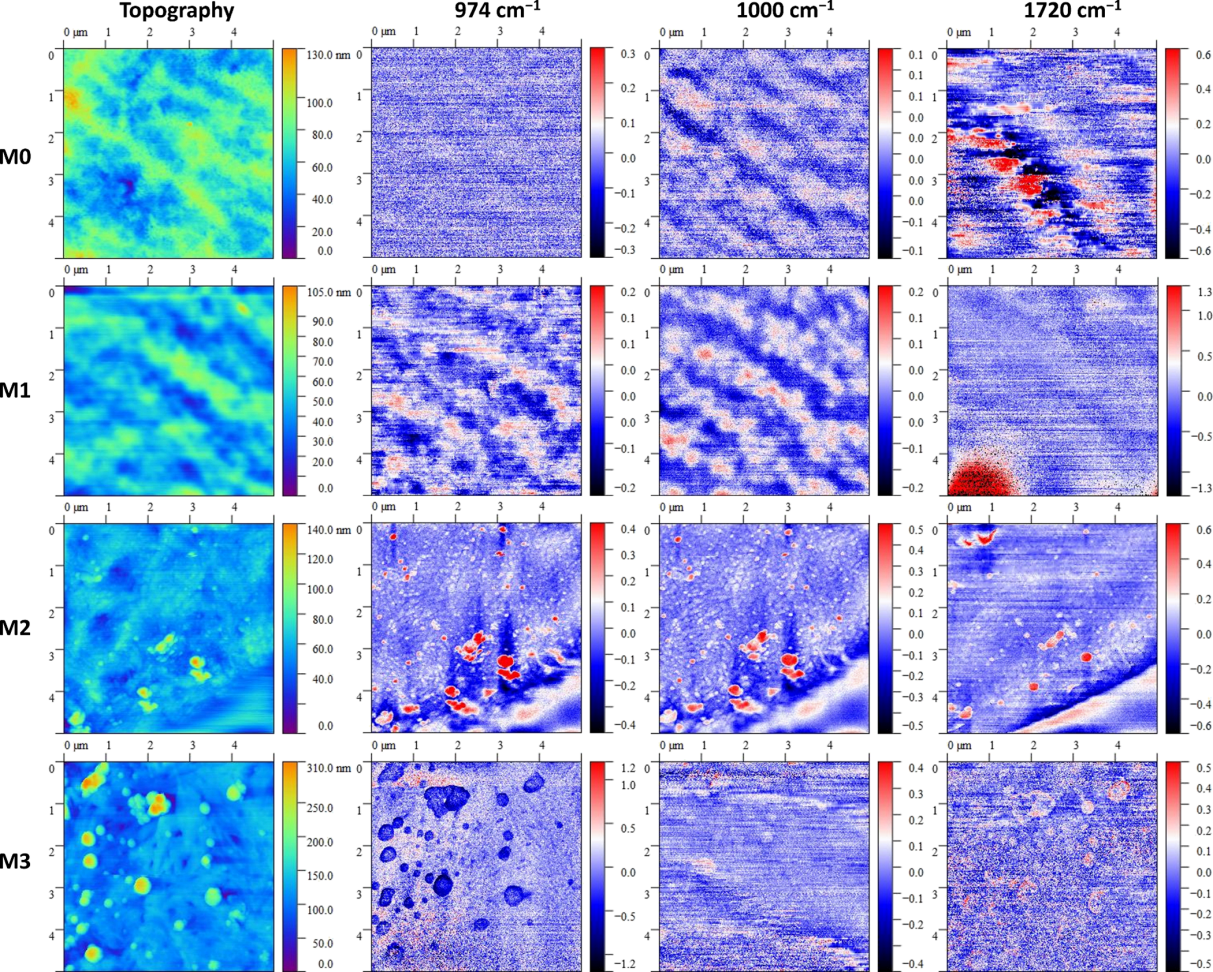
2D images of data from IR-sSNOM analysis with PVDF membranes. The surface topography images obtained with AFM (left) and the optical channel image of the O2P at excitation wavenumbers of 974 cm^–1^ (second column), 1000 cm^–1^ (third column), and 1720 cm^–1^ (fourth column) are shown. Each row represents a different membrane sample (from M0 at the top to M3 at the bottom) with different QDMA and MPC ratios.

Figure 5 shows the AFM topography and optical phase records demodulated at the second harmonic frequency of the tip oscillation (O2P) for all of the measured samples. For the wavenumbers that are characteristic of PVDF (974 and 1000 cm^–1^), the O2P channel shows a relatively homogeneous area on almost all samples, indicating that the sample is formed by a compact and coherent polymer layer. While no significant features are visible on the M0 and M1 membranes, very distinct features were formed on the other membranes. Membrane M1 has a very significant contrast in the left corner at 1720 cm^–1^. This was not caused by the interaction between incident light and chemical entities, but this signal was the result of missing pixels in the image. In the M2 sample image, a bright area could be observed in the lower right corner on all collected O2P channels, which is caused by the tip falling out of contact with the membrane. For some of the images, a higher noise level was observed, which is due to the fact that a very low excitation laser intensity had to be used for their measurement, e.g., the O2P images at 1000 and 1720 cm^–1^ for the M3 sample. It is apparent that the modification of the membranes by the addition of QDMA and/or MPC side chains led to a change in the optical properties of the membranes, which caused them to strongly absorb IR radiation in certain regions. The high intensity of the laser would therefore lead to a deformation and thermal decomposition of the sample, which would degrade the sample at that location. At the same time, the membrane decomposition releases substances that can stick to the tip, which becomes unusable for further measurements. Therefore, it was necessary to finely tune the excitation radiation intensity for all samples to be sufficient for signal observation but safe for both the sample and the tip at the same time. The last irradiation wavenumber, at which the samples were analyzed, was 1720 cm^–1^, which is attributed to the C=O bonds on the side chain of the MPC and QDMA additives. There is observable contrast in the O2P channel images, which is largely due to the morphology of the membrane; however, there are also changes in chemical signals in regions of aggregates’ presence and other morphological changes on the samples M2 and M3. This observation suggests that increasing the molar fraction of MPC in attached side chains can lead to the formation of aggregates containing higher concentrations of MPC or QDMA side chains. As the temperature changes during the measurements, there is a slight positional shift in some of the measured images, mainly due to the thermal expansion of the membrane (thermal drift), as may be seen, for example, for sample M2 in Figure 5.

### 3.3 Raman Microscopy

**Figure 6:**
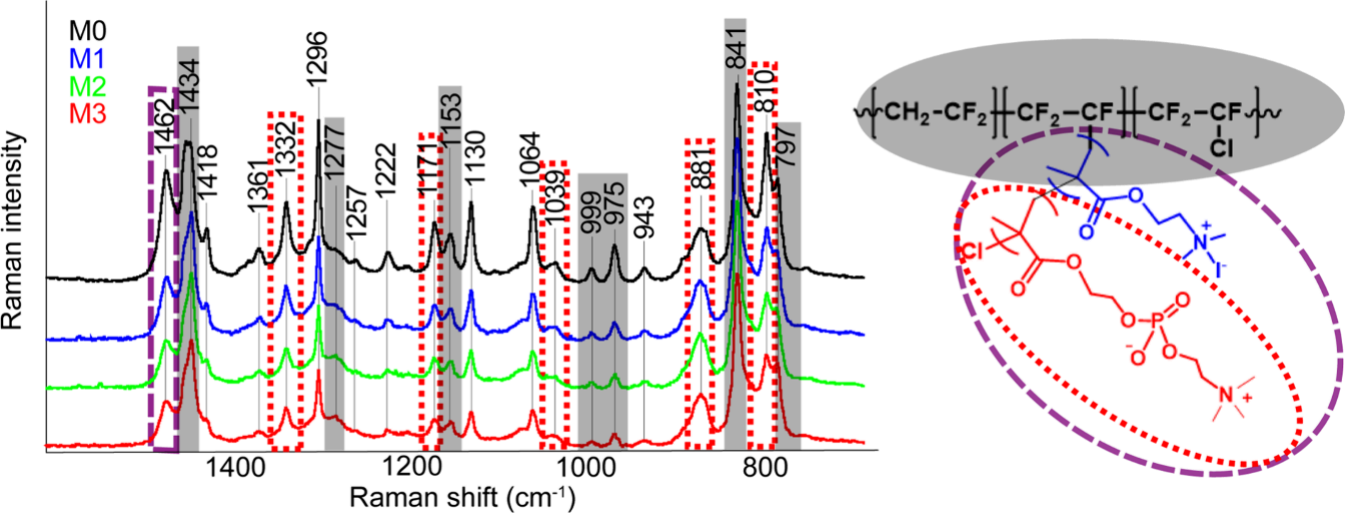
Averaged Raman spectra of the PVDF membrane samples (left, from top to bottom: M0, M1, M2, and M3) and the structure of the polymer with color assignment (right). The highlighted spectral regions correspond to vibrations of the main PVDF chain (gray), the MPC side chain (red dots), or shared bands for both side chains (violet lines).

As Raman spectroscopy provides complementary information to FTIR spectroscopy, it was utilized to better understand the chemical structure of the studied samples. Furthermore, the bands in Raman spectra are typically sharper and more distinct than those in Micro-FTIR spectra, which allows for an easier fitting of the bands. The Raman spectra of the PVDF samples are presented in Figure 6 in the same way as the Micro-FTIR spectra. The next advantage of Raman microscopy was the range of the used detector (3200–100 cm^–1^), which allowed us to collect additional information from the region of lower wavenumbers, where typical bands of the PVDF’s crystalline phases are located. Based on this information, it was possible to depict spectral maps based on the peaks’ intensity, which is crucial for finding out the distribution of different PVDF phases across the surface of the studied samples.

In all averaged Raman spectra, the vibrational bands were assigned to the main PVDF chain as well as bands corresponding to groups on the side chains, some of which are bands composed of multiple overlapping vibrations. The results of subsequent mathematical fitting of those bands can be viewed in the Supporting Information (Figure S2). The presence of such complex bands indicates the complexity of the structure of the copolymer samples. The assignment of the bands to the specific vibrations is difficult because of the presence of the same functional groups, e.g. CH_2_, at several locations in both the main and side chains.

In the area of the band characteristic of CH_2_ groups at ca. 1450 cm^–1^, numerous contributing bands were detected in the Raman spectra. These single bands were assigned to the scissoring vibrations of the CH_2_ group in the aliphatic part, while the lower part of the band is assigned to the same group next to the electronegative element, namely, fluorine and/or carbonyl. The band at 1277 cm^–1^ corresponds to the coupled stretching symmetric vibration of the CF_2_ and C–C groups and the skeletal C–C–C vibration. The band at 1153 cm^–1^ consists of contributions from two vibrations, namely, the antisymmetric stretching C–C vibration and the symmetric stretching CF_2_ group vibration. The band at 999 cm^–1^ is assigned to the skeletal F–C–Cl vibration, which is located on the main chain of the modified PVDF. The band at 975 cm^–1^ can be identified as the CH_2_ group’s torsional vibration. The lowest investigated band is at 797 cm^–1^, which originates from the CH_2_ group vibration.

The band at 1462 cm^–1^ is the first displayed band, which consists of two different CH_2_ group vibrations. The first maximum is at 1463 cm^–1^, and the second much smaller maximum is at 1470 cm^–1^. Both modes are located out of the electronegative element, but this indicates that the polymer chain is not uniform (Figure S2A). The stretching vibrations of the P=O group and the C–F group contribute to a significant peak at 1332 cm^–1^, where the C–F vibration plays the main role (Figure S2B). The band at 1171 cm^–1^ is wide, and the band's fitting revealed that this peak consists of three contributions from different groups (mainly CH_2_ and P=O) (Figure S2C). The rocking vibration of the CH_2_ group from the connection between the side chains and the main chain of PVDF has different vibrations due to the neighboring electronegative atoms. The next contribution from the P=O group is manifested as a stretching vibration at 1039 cm^–1^. The fitting of this band revealed that it also contains two overlapping vibrations. The first one arises from the P–O–C stretching vibration, and the second one belongs to the C–C stretching vibration (Figure S2D). The band at 881 cm^–1^ is the result of the overlapping antisymmetric stretching of the C–C group, the symmetric stretching of the CF_2_ group, and the PO_4_ symmetrical stretching vibration (Figure S2E). The band at 841 cm^–1^ is common to the rocking vibration of the CH_2_ group and the stretching antisymmetric vibration of the CF_2_ group. The phosphate group also exhibits a P=O stretching vibration, which is present at 810 cm^–1^; however, this band also contains contributions from the PVDF γ crystalline phase and the rocking vibration of the CH_2_ group (Figure S2F). This peak is the most significant for sample M0 because of the increased presence of the γ crystalline phase; however, for the modified membranes, the bands of this phase are suppressed. The assignment of all vibrations in the Raman spectra is summarized in Table S2 in the Supporting Information.

**Figure 7:**
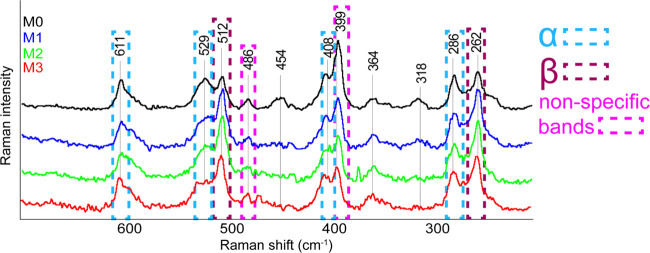
Details of the averaged Raman spectra of PVDF membrane samples (M0, M1, M2, and M3) with marked crystalline modifications. The highlighted spectral regions correspond to vibrations of the α (blue) and β (violet) crystalline phases of PVDF or to the nonspecific bands (pink) of the polymer.

The next advantage of Raman microscopy was the mentioned range of the used detector, which allows us to obtain additional information from the region of lower wavenumbers, where typical bands for the PVDF’s crystalline phases are located (Figure 7). Based on this information, it was possible to depict spectral maps based on the peaks’ intensity, which is crucial for finding out the distribution of different PVDF phases across the surface of the studied samples. The bands that are typical for the α phase are at 975 and 797 cm^–1^, while for the β phase, they are at 1279 and 881 cm^–1^. A common band for both the β and γ phases is at 841 cm^–1^, and the typical band for the γ phase only is at 810 cm^–1^, which appears in the spectra only as a shoulder band.^43^ Larger differences between the phases can be found in the lower wavenumber regions, which are highlighted in Figure 7. The bands for the α, β, and γ phases have been defined based on the articles by Martins et al. and Constantino et al.^42,43^

Figure 7 shows the region of the lower Raman shifts in detail. In this spectral region, the α and β crystalline phases of PVDF are clearly distinguishable from each other. The band at 611 cm^–1^ belongs to the scissoring vibration of the CF_2_ group and the C–C–C group of the α phase. Another band that can be assigned to the α phase is at 529 cm^–1^ originating from the scissoring vibration of the CF_2_ group. The 512 cm^–1^ band belongs to the scissoring vibration of the CF_2_ group of the β phase. The band at 486 cm^–1^ may be identified as the rocking vibration of the CF_2_ group. The band at 408 cm^–1^ arises from the rocking vibration of the CF_2_ group in the α phase. The 399 cm^–1^ band can then be assigned to the deformation vibration of the C–Cl group, which is again located in the main polymer chain. The band at 286 cm^–1^ belongs to the torsional and rocking vibration of the CF_2_ group and is related to the α phase. The last visible band is at 262 cm^–1^, which is interpreted in the literature as a torsional vibration of the CF_2_ group belonging to the β phase.^43^ The assignment of all vibrations in the lower Raman shift range is summarized in Table S3 in the Supporting Information.

#### 3.3.1 Raman Mapping

Raman spectral mapping is a perfect tool to distinguish areas with different chemical properties. The Raman mapping provided relatively similar information to IR-sSNOM. On the one hand, the IR-sSNOM imaging has a much better lateral resolution (10–20 nm) due to surpassing Abbe's diffraction limit^17^ by the utilization of the AFM tip and detection of a near-field signal. On the other hand, the Raman mapping is able to provide the whole Raman spectra in each measured point and, furthermore, in the region of low wavenumbers, unlike the IR-sSNOM, which only enables the detection of optical contrast at the selected irradiation wavenumber within the available ranges of the QCL lasers.

**Figure 8:**
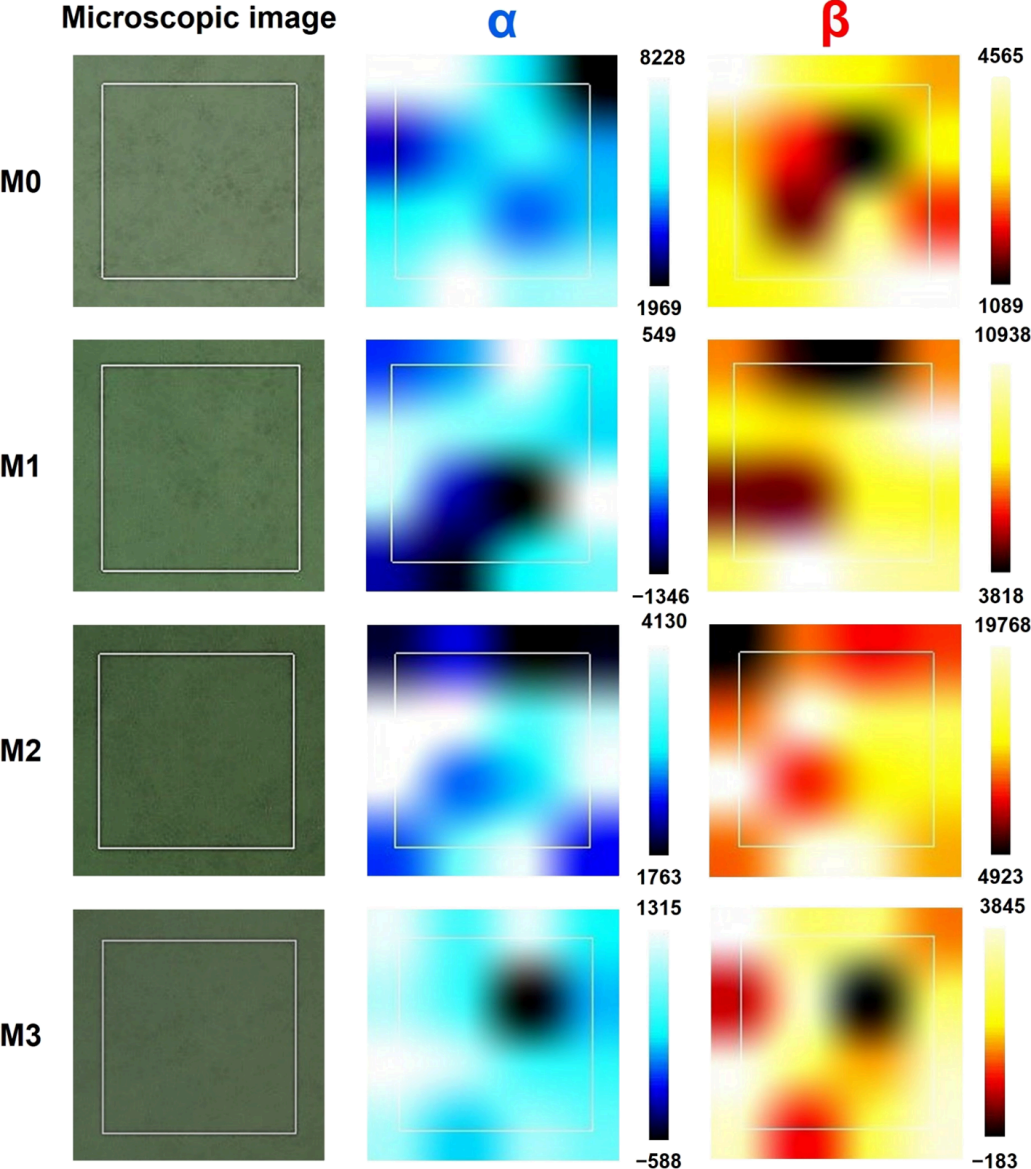
Raman microscopic mapping of a 4 × 4 μm area on PVDF membrane samples. Microscopic images at 100× magnification (left) and spectral maps (4 × 4 points) displaying intensity difference at Raman shifts of 797 cm^–1^ (corresponding to the α crystalline phase of PVDF, middle) and at 881 cm^–1^ (corresponding to the β crystalline phase of PVDF, right).

In Figure 8, a microscopic view of the individual membranes is displayed in the left column. The white squares represent the analyzed area. Using microscopic images, it can be confirmed that the surface of M1 and M2 samples is morphologically homogeneous at the microscale without the presence of large aggregates or fibers. Slightly increased roughness may be observed for the M0 and M3 membranes, which may be the reason for the observed differences between the collected spectra. At first sight, the differences in the scale (i.e., peak intensity) for the α and β phases can be seen in Figure 8, which is due to the fact that the 881 cm^–1^ band, characteristic of the β phase, is typically more intense. The reason for such a high intensity when compared to the α phase is the contribution of several vibrations to this mode. There is almost no difference between samples M0 to M2, but more homogeneous detected high band intensity can be seen for sample M3. This is due to the highest content of MPC salt with the phosphate group, whose vibration is located at a very similar position, as the discussed β phase band. The results show that the crystalline α and β phases of the PVDF polymer are not homogeneously distributed over the membrane surface but rather concentrated in separate sites. For almost all samples, the most intense locations of the individual crystalline phases are directly near each other or only slightly overlapping. Each scanned area has a maximum of two high spectral intensity areas, and the rest of the scan shows almost homogeneous intensity. Thus, it can be stated that the crystalline phase changes freely and abruptly between the two types of studied phases. Negative values on the axes are caused by adjusting the color scale. Furthermore, the results show that the areas with a minimum occurrence of one phase exhibit a maximum spectral intensity of the other phase. Additionally, Raman maps of the lower Raman shifts are displayed in Figure S4. Generally, both maps show similar results, although slight differences can be observed. These differences are probably caused by the presence of overlapping bands, but this does not disturb the qualitative importance of these results.

## 4 Conclusions

This study aimed to form a suitable methodology for modified PVDF membrane characterization concerning their macroscopic and nanoscopic properties. For such purpose, the combination of various microscopic and nanoscopic techniques was utilized, taking into account each method’s strengths and weaknesses when facing the need to record changes induced by the membrane’s modification by the addition of QDMA and MPC side chains. Raman and infrared microscopies proved to be able to detect the presence of modifying side chains despite the very low concentration of both of them. Identification of the C=O group, which can be found in both side chains, and observation of the increased width of the bands typical for CH_2_ groups, whose position is strongly affected by the proximity of electronegative elements, were the most evident manifestations of the side chains’ presence.

The PVDF polymer is often present in three different crystalline phases, which exhibit different spectral signatures, especially in Raman spectra at lower vibration energies.^42,43^ These three crystalline phases, α, β, and γ, were observed in the Raman spectra, while α and β are spectroscopically very well distinguishable. Therefore, Raman spectral mapping was performed to monitor the distribution of the α and β phases. Based on these measurements, the occurrence of the crystalline phases was investigated, leading to find that both the α and β crystalline phases are present on the surface and that they occur independently of each other.

The last technique implemented was IR-sSNOM, which allows for nanometer spatial resolution. This technique not only revealed the properties that can be inferred from the topography of individual membranes, such as grain height, number of grains per area, and roughness, but also proved to be worthy while exploring the abundance of particular aggregates of varying chemical origin. It was simultaneously determined that M2 and M3 membranes contain surface adherent features, while the surfaces of M0 and M1 membranes are almost uniform. Aiming to investigate the surface distribution of modifying side chains, we selected a wavenumber characteristic of both materials (MPC and QDMA). Membranes M2 and M3 exhibited a corresponding chemical signal at the aggregates; therefore, it is clear that the aggregation occurs with an increasing molar fraction of MPC in the side chains. To conclude, all employed techniques proved to be able to provide valuable information about the membrane samples, while most of them would not have been obtainable by other commonly used surface characterization methods.

## Supporting Information Available

Setup scheme, peak resolving, Raman/IR spectra, and vibrational band assignment (PDF)
