# Supplementary material for: Characterization of Modified PVDF Membranes Using Fourier Transform Infrared and Raman Microscopy and Infrared Nanoimaging: Challenges and Advantages of Individual Methods
Source: ACS Omega. 2024 May 31;9(23):24685–94. doi: 10.1021/acsomega.4c01197 (PMC11170652; doi:10.1021/acsomega.4c01197)
Supplement: Supplementary file 1 — ao4c01197_si_001.pdf [file ao4c01197_si_001.pdf]

Supplementary Information

Characterization of Modified PVDF Membranes using FTIR and Raman microscopy and IR nanoimaging – Challenges and Advantages of Individual Methods

Matěj Kmetík*, Ivan Kopal, Martin Král, Marcela Dendisová

Department of Physical Chemistry, University of Chemistry and Technology Prague, Technická 5, 166 28 Prague 6, Czech Republic

* Corresponding author: tel.: + 420 220 444 040,

e-mail: kmetikm@vscht.cz

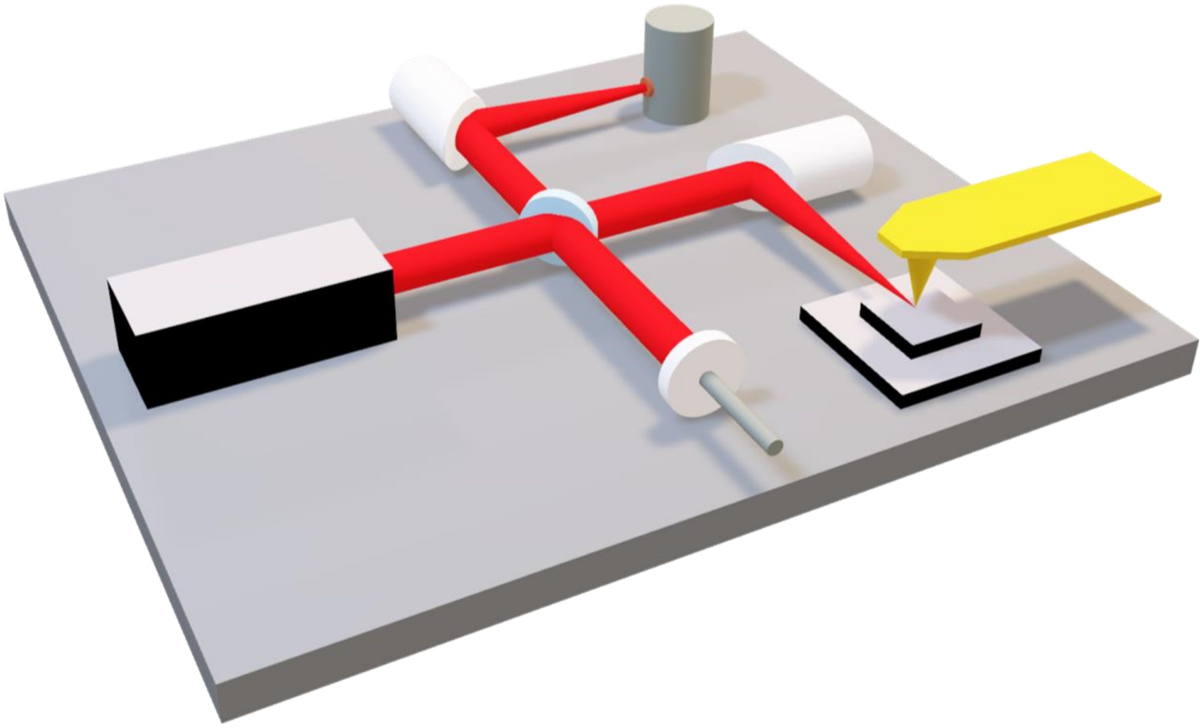

Figure S1 **Scheme of inner parts of IR-sSNOM.** The red color presents the laser path. The yellow part is the AFM tip in tapping mode. The source of radiation is the quantum cascade tunable laser. The middle part of the instrument is the Michelson interferometer, and the final part is the MCT detector in the upper right corner.

Several bands in the Raman spectra have been fit by profile functions so that the positions of the individual bands (shoulders) contributing to them can be better distinguished. All the presented peak resolved data were fitted using the Voigt method available within the Omnic software (ver. 9.12.928).

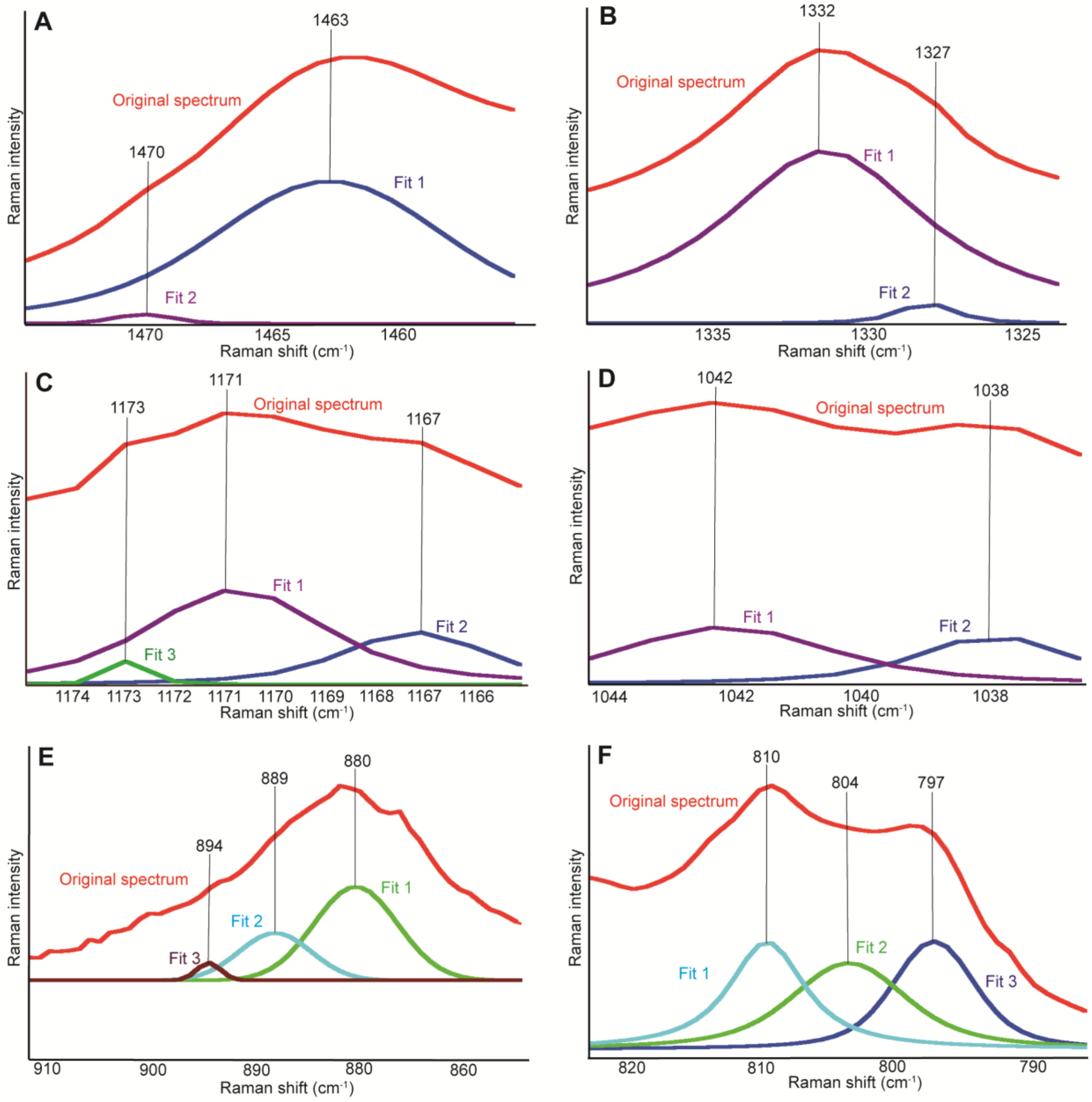

Figure S2 **Peak resolution of six different regions (A–F) of Raman spectra.**

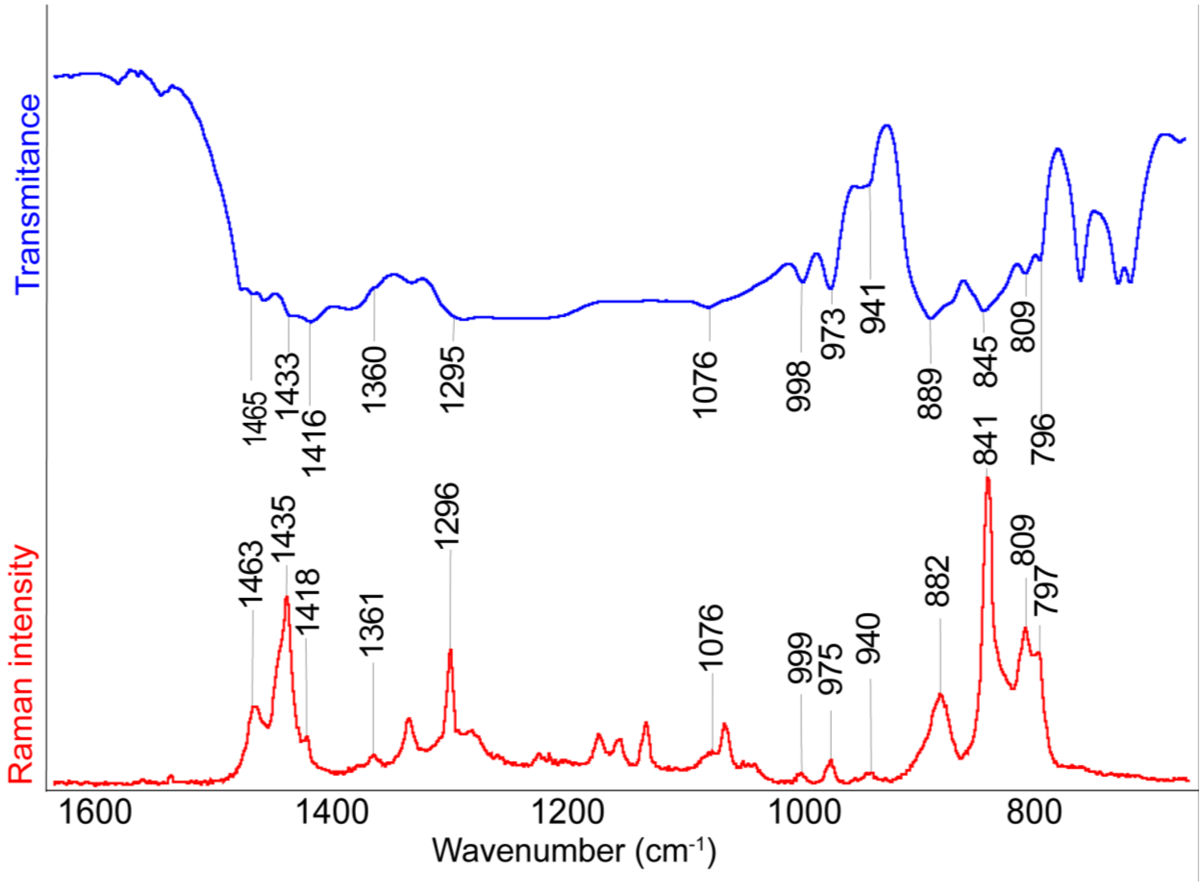

Figure S3 **Comparison of membrane M2 spectra measured by Raman microscopy and Micro-FTIR spectroscopy.**

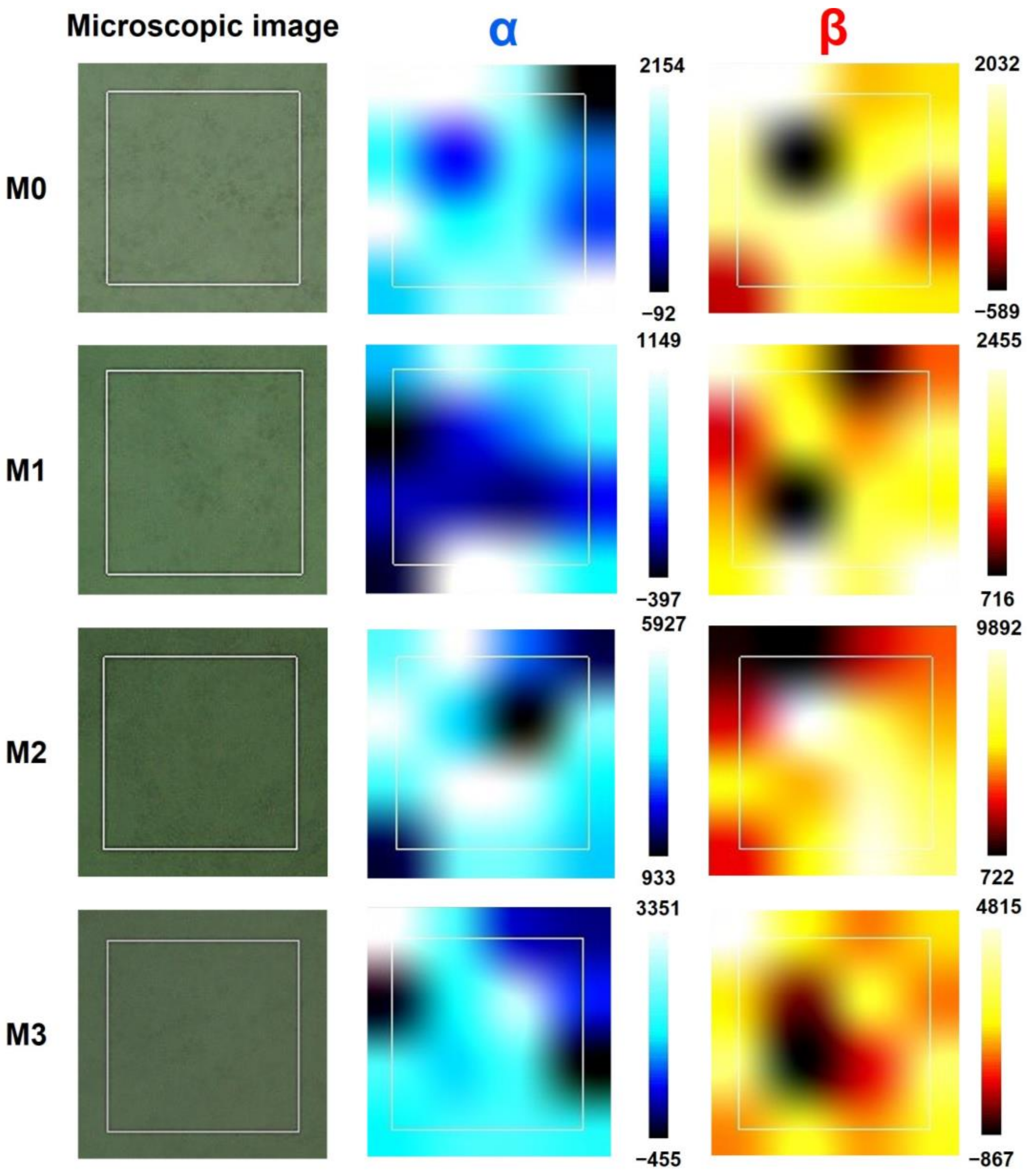

Figure S4 Raman microscopic mapping of 4 × 4 μm area on PVDF membrane samples. Microscopic images at 100× magnification (left), spectral maps (4 × 4 points) displaying intensity difference at a Raman shift of 611 cm^{-1} (corresponding to the α crystalline phase of PVDF, middle) and at 262 cm^{-1} (corresponding to the β crystalline phase of PVDF, right).

Table S1 Assignment of vibrational bands in Micro-FTIR spectra of modified PVDF [1].

Wavenumber (cm^{-1})	Group	Vibration type	Origin
1720	C=O	Stretching	Both side chains
1540	CH_2	Stretching	Main chain
1464	CH_2	Stretching	Both side chains
1453	CH_2	Stretching	Main chain
1329	P=O	Stretching	MPC
1284	CF_2	Stretching	Main chain
1164	P=O	Stretching	MPC
1000	F–C–Cl	Skeletal	Main chain
974	CH_2	Twisting	Main chain
889	C–C	Stretching	Main chain
843	CH_2	Rocking	Main chain
809	P=O	Stretching	MPC
798	CH_2	Rocking	Main chain
763	CF_2	Rocking	Main chain

Table S2 Assignment of vibrational bands in Raman microscopy spectra of modified PVDF [2].

Wavenumber (cm^{-1})	Group	Vibration type	Origin
1462	CH_2	Stretching	Both side chains
1434	CH_2	Stretching	Main chain
1332	P=O, C–F	Stretching, Stretching	MPC
1277	CF_2, C–C, C–C–C	Stretching, Stretching, Skeletal	Main chain
1171	CH_2, P=O	Rocking, Stretching	MPC
1153	C–C, CF_2	Stretching	Main chain
1039	P=O	Stretching	MPC
999	F–C–Cl	Skeletal	Main chain
975	CH_2	Twisting	Main chain
881	C–C, CF_2, PO_4	Stretching, Stretching, Stretching	MPC
841	CH_2, CF_2	Rocking, Stretching	Main chain
810	P=O, CH_2	Stretching, Rocking	MPC
797	CH_2	Rocking	Main chain

Table S3 Assignment of vibrational bands of PVDF and its´ crystalline phases in the lower Raman shift region [2].

Raman shift (cm^{-1})	Group	Vibration type	Phase
611	C–C–C, CF_2	Stretching, Scissoring	α
529	CF_2	Scissoring	α
512	CF_2	Scissoring	β
486	CF_2	Rocking	Non-specific
408	CF_2	Rocking	α
399	C–Cl	Deformation	Non-specific
286	CF_2	Torsional, Rocking	α
262	CF_2	Torsional	β

References

(1) Martins, P.; Lopes, A. C.; Lanceros-Mendez, S. Electroactive phases of poly(vinylidene fluoride): Determination, processing and applications. *Progress in Polymer Science* **2014**, *39* (4), 683-706. DOI: 10.1016/j.progpolymsci.2013.07.006.

(2) Constantino, C. J. L.; Job, A. E.; Simões, R. D.; et al. Phase Transition in Poly(Vinylidene Fluoride) Investigated with Micro-Raman Spectroscopy. *Applied Spectroscopy* **2005**, *59* (3), 275-279. DOI: 10.1366/0003702053585336.
